# Comparative Reactive Oxygen Species (ROS) Content among Various Flavored Disposable Vape Bars, including Cool (Iced) Flavored Bars

**DOI:** 10.3390/toxics9100235

**Published:** 2021-09-25

**Authors:** Shaiesh Yogeswaran, Thivanka Muthumalage, Irfan Rahman

**Affiliations:** Department of Environmental Medicine, University of Rochester Medical Center, Box 850, 601 Elmwood Avenue, Rochester, NY 14642, USA; Shaiesh_Yogeswaran@urmc.rochester.edu (S.Y.); Thivanka_Muthumalage@urmc.rochester.edu (T.M.)

**Keywords:** vaping, ENDS, disposable e-cigarettes, vape bars, flavoring, flavoring chemicals, reactive oxygen species (ROS), disposables, oxidative stress

## Abstract

Studies have shown that aerosols generated from flavored e-cigarettes contain Reactive Oxygen Species (ROS), promoting oxidative stress-induced damage within pulmonary cells. Our lab investigated the ROS content of e-cigarette vapor generated from disposable flavored e-cigarettes (vape bars) with and without nicotine. Specifically, we analyzed vape bars belonging to multiple flavor categories (Tobacco, Minty Fruit, Fruity, Minty/Cool (Iced), Desserts, and Drinks/Beverages) manufactured by various vendors and of different nicotine concentrations (0–6.8%). Aerosols from these vape bars were generated via a single puff aerosol generator; these aerosols were then individually bubbled through a fluorogenic solution to semi-quantify ROS generated by these bars in H_2_O_2_ equivalents. We compared the ROS levels generated by each vape bar as an indirect determinant of their potential to induce oxidative stress. Our results showed that ROS concentration (μM) within aerosols produced from these vape bars varied significantly among different flavored vape bars and identically flavored vape bars with varying nicotine concentrations. Furthermore, our results suggest that flavoring chemicals and nicotine play a differential role in generating ROS production in vape bar aerosols. Our study provides insight into the differential health effects of flavored vape bars, in particular cool (iced) flavors, and the need for their regulation.

## 1. Introduction

Despite the significant decline in youth e-cigarette usage since the Federal Drug Enforcement Agency’s (FDA) flavored e-cigarette enforcement policy which was enacted in February 2020, youth e-cigarette use within the United States remains significantly high [1]. Moreover, according to a cross-sectional study conducted by the Centers for Disease Control and Prevention (CDC), in 2020, 4.7% of middle school students (550,000) and 19.6% of high school students (3.02 million) reported current e-cigarette use [1]. The prevalence of e-cigarette usage in the United States, especially amongst its youth, is partly due to the switch many cartridge-based e-cigarette users made to using disposable e-cigarettes; the FDA’s 2020 e-cigarette flavoring enforcement policy prompted this action [1]. Further, the FDA’s flavoring enforcement policy only applies to flavoring for cartridge-based Electronic Nicotine Delivery System (ENDS) products; these products include cartridge-based e-cigarettes and pre-filled pod devices [1]. More specifically, the FDA’s February 6th, 2020 e-cigarette enforcement policy for cartridge-based ENDS products applies to all flavors with nicotine, excluding menthol and tobacco [1]. Moreover, the FDA’s enforcement policy involves requiring all manufacturers and retailers in the United States to remove all flavored cartridge-based ENDS products with nicotine from the market except tobacco-flavored and menthol-flavored cartridge-based ENDS products [1]. All flavored products without nicotine (zero nicotine) are still available in the market. Furthermore, products exempt from the previously mentioned enforcement policy include disposable e-cigarettes with or without nicotine in certain states within the United States. A disposable e-cigarette is a type of ENDS product which can be discarded or thrown away once it runs out of e-liquid or charge. According to the 2020 National Youth Tobacco Survey (NYTS) conducted by the CDC, the use of disposable e-cigarettes (e-cigs) by high-school students who were already e-cig users had increased significantly from 2.4% in 2019 to 26.5% in 2020. Additionally, according to the 2020 NYTS, the number of middle-school e-cig users who specifically used disposable e-cigs increased from 3.3% in 2019 to 15.2% in 2020 [1]. One aspect of disposable e-cigs which is attractive to youth e-cigarette users is the convenience at which they can be used; they do not require recharging or refilling with e-liquids like cartridge-based products. Additionally, disposable devices are much cheaper and practical to use than their refillable counterparts.

With the substantial rise in the availability of different e-liquid flavors in recent years, investigating the role that e-liquid flavoring chemicals have in inducing pulmonary pathophysiological effects has become more complicated [2]. Further, the long-term effects of e-cigarette vapor exposure on human health require further investigation. However, studies so far have shown that e-cigarette aerosol production involves generating reactive oxygen species (ROS) [3]. ROS can be generated either intracellularly (via mitochondrial oxidative phosphorylation) or may arise from exogenous sources (cigarette smoke, e-cigarette aerosols, and environmental pollution,) [4]. Specific ROS include hydrogen peroxide (H_2_O_2_), hydroxyl radical (^•^OH), and superoxide radical (O_2_^•−^) [5]. ROS plays a crucial role in modulating the immune-inflammatory system and activating different signal transduction pathways and cell signaling processes for inflammatory responses [6].

The normal physiological balance between ROS and antioxidants can be disturbed through the inhalation of exogenous sources of ROS, thus leading to the damage of cellular structures. Further, an excess in intracellular ROS levels causes oxidative damage to the cellular membrane, intracellular lipids, intracellular enzymes, and intracellular DNA (iDNA). Moreover, excess ROS can also induce a vicious cycle of chronic inflammation in the lungs due to excessive ROS leading to the activation of specific immune cells, polymorphonuclear neutrophils (PMNs); activated PMNs can, in turn, generate more ROS in pulmonary cells [7]. This subsequent chronic inflammation leads to airways becoming more thickened and prone to mucus secretion, also known as airway modeling, this later resulting in lung dysfunction [8]. Regarding exogenous ROS sources, studies in the past have shown that tobacco smoke-generated ROS can induce DNA damage within lung epithelial cells and premature pulmonary cell death, leading to the development of lung cancer and COPD/emphysema, respectively [9]. Additionally, one study had shown that through activating the heating element of an e-cigarette and then aerosolizing its e-liquid component, ROS is produced; which can be drawn from the device into the lungs, directly causing inflammatory response [10].

Despite the well-known adverse health effects of conventional cigarette smoking, one of the main factors driving both youth and adult appeal for e-cigarettes is the availability of many different flavors. These flavors add to the allure many have for e-cigarettes by creating sensory perceptions of palatable tastes, which conceal the bitter taste of nicotine [11]. Further, one survey found that the availability of fruit and candy e-liquid flavors significantly contributes to the prevalence of youth e-cigarette usage in the United States; adults seem to prefer more traditional flavors, such as tobacco [11]. Likewise, according to a Morbidity and Mortality Weekly Report by the CDC conducted in September 2020, among current users of flavored disposable e-cigarettes, the most commonly used flavor type was those under the fruit classification (82.7%; 650,000 [1]). Additionally, according to the same Morbidity and Mortality Weekly Report by the CDC, the following three most widely used vape bar flavors were those falling under the mint classification (51.9%; 410,000), those falling under the sweet categorizations (candy, desserts, etc.) (41.7%; 330,000), and those falling under the menthol (cool/iced) classification (23.3%; 180,000), respectively [1]. Accordingly, with the recent surge in flavored disposable e-cig use during this past year, more research should be conducted which investigates how ROS content within aerosols generated from disposable e-cigarettes are modulated by flavoring chemicals.

In addition to flavor, another factor contributing to the prevalence of disposable e-cigarette usage in this country is the range of nicotine concentrations which are available for these devices. Nicotine is a highly addictive alkaloid present within the aerosol generated by e-cigarettes as well as within the smoke generated from conventional cigarettes [12]. For disposable e-cigarettes sold within the United States, nicotine content ranges from 0 mg/mL (0%, nicotine-free option) to 68 mg/mL (6.8%). Furthermore, nicotine is extremely addictive and can harm the neural development of those under the age of 25, which is most troubling given the prevalence of e-cigarette use among adolescents in this country [13]. Exposure to nicotine through inhaling e-cigarette generated aerosols has contributed to prolonging e-cigarette usage amongst a significant portion of the country, especially those under the age of 25 [14]. Despite youth e-cigarette usage continuing to be a rising health concern in the U.S, studies investigating how exogenous ROS generation varies as a function of nicotine concentration in ENDS products are lacking. Additionally, with the recent surge in flavored disposable e-cig use and the wide range of nicotine content available for these products, research should be conducted to determine how ROS or free radical generation among disposable e-cigarettes varies as a function of nicotine concentration. Consequently, in our study, we hypothesize that ROS levels within the aerosols generated from disposable e-cigarettes will vary with different flavors as well with different nicotine concentrations. Furthermore, disposable e-cigarettes with a wide range of salt nicotine concentrations (0–6.8%) and within six main flavor categories (Tobacco, Minty Fruit, Fruity, Minty/Cool (Iced), Desserts, and Drinks/Beverages) from different vendors were analyzed. Additionally, we analyzed vape bars of identical flavors manufactured from the same company, but with varying concentrations of nicotine. The company (vendor) that produced these bars that we subsequently analyzed were Bolt, Flair Plus, and SMOQ. Bolt and Flair Plus disposable bars, which contain a solution comprising Propylene Glycol (PG) and Vegetable Glycerin (VG) mixed in a 1:1 ratio; likewise, these bars use a 1.6 and 1.8 Ohm coil, respectively, to aerosolize their component e-liquid. Accordingly, our subsequent comparative acellular ROS analyses included semi-quantified ROS content within aerosols produced from our PG:VG controls heated using 1.6 and 1.8 Ohm coils; the controls were made using a 1:1 (i.e., 50:50 ratio) ratio of PG and VG in this pilot/preliminary screening study.

## 2. Materials and Methods

### 2.1. Vape Bar Procurement

Vape bars were purchased from various locations and manufacturers locally within Rochester, NY and from various online websites/vendors. The disposable e-cigarettes used in this experiment contained a wide range of salt nicotine concentrations (0–6.8%) and were categorized into six main flavor categories (Tobacco, Minty Fruit, Fruity, Minty/Cool (Iced), Desserts, and Drinks/Beverages). The commercial manufacturers of the disposable vape bars used were Blu, Bolt, Cyclone, Eonsmoke, Flair Plus, Fling, Fliq, FreshBar, Hyde, Hyppe Bar, Jolly, Lit, NJOY, Phantom, Puff Bar, SMOQ, SOL, Tsunami Twin, Vice, Zaero, and Zero Disposable.

### 2.2. Generation of Vape Bar Aerosols

A fluorogenic dye was made using 0.01N NaOH, 2′7′ dichlorofluorescein diacetate (H_2_DCF-DA) (EMD Biosciences, San Diego, CA, USA) (Cat # 287810), phosphate (PO_4_) buffer, and horseradish peroxidase (Thermo Fisher Scientific, Waltham, MA, USA (Cat# 31491). The PO_4_ buffer was made using dibasic sodium phosphate (Sigma-Aldrich, St. Louis, MO, USA) (Cat# S0876) and sodium phosphate monobasic (JT Baker, Phillipsburg, NJ, USA) (Cat # 02-004-215). Afterward, i.e., upon bubbling, the resulting fluorogenic dye was analyzed via fluorescence spectroscopy with a maximum excitation and emission spectra of 475 and 535 nm, respectively. The standards used in this experiment ranged from 0 to 50 μM, each made from 1.25 mM H_2_O_2_ solution, which was prepared from 30% H_2_O_2_ (H_2_O_2_) (Thermo Fischer Scientific, Waltham, MA, USA) (Cat# H323-500) and double-distilled water (ddH_2_O). To enumerate, 1.25 mM H_2_O_2_ was diluted to 0.90 mM H_2_O_2_ using ddH_2_O, and that resulting 0.90 mM hydrogen peroxide solution was used in preparing the previously mentioned standards. Further, to ensure the desired concentration of H_2_O_2_ had been prepared using the 30% H_2_O_2_ solution (1.25 mM), Ultraviolet/Visible (UV/Vis) spectroscopy was used. To further explain, after adding 113 μL of 30% H_2_O_2_ to 999.887 mL of ddH_2_O, 1 mL of the resulting solution was added to a quartz cuvette (Sigma-Aldrich, St. Louis, MO, USA) (Cat # C-9542). The quartz cuvette, which has a 10 mm light path, was then inserted into a UV/Vis Spectrophotometer (Beckman Colter, Brea, CA, USA) (Cat# DU 250) and exposed to 240 nm light. Afterward, the absorbance was read and divided by 0.0436 (the extinction coefficient); the extinction coefficient was determined through previous H_2_O_2_ standardization tests and calibration curves generated. The resulting calculation should produce 1.25 mM; this signifying the correct concentration of H_2_O_2_ was produced using double distilled water and 30% H_2_O_2_. Further, before adding 1 mL of the resulting H_2_O_2_ solution into a quartz cuvette to then be inserted into the UV/Vis Spectrophotometer, ddH_2_O was pipetted into the same quartz cuvette and used as a blank.

**Figure 1:**
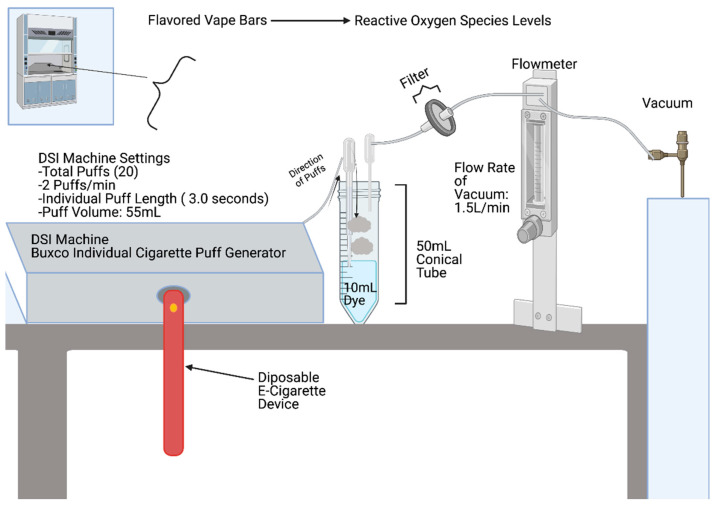
Disposable E-cigarette exposure generation system. This schematic shows the apparatus used to bubble the 10 mL fluorogenic dye within each 50 mL conical tube using the aerosol emitted from the vape bar inserted into the DSI Puff Generator machine. Using a standard lab vacuum, the fluorogenic dye was bubbled at 1.5 L/min, and “puffs” were generated from each vape bar using the DSI Machine above. The DSI machine provided a total of 20 puffs, each puff lasting three seconds and having a volume of 55.0 mL. Each conical tube was wrapped in aluminum foil to protect the fluorogenic dye from light. The entirety of the “bubbling” process using the DSI machine and vacuum apparatus was performed inside a chemical fume hood.

Regarding the puff generation protocol itself, using a standard lab vacuum and a Buxco Individual Cigarette Puff Generator (Data Sciences International (DSI), St. Paul, MN, USA) (Cat#601-2055-001), the aerosol generated from each vape bar was individually bubbled through 10 mL of H_2_DCF-DA solution within a 50 mL conical tube, at 1.5 L/min (Figure 1). Moreover, two lime glass Pasteur pipettes (VWR, Radnor, PA, USA) (Cat # 14672-380) were inserted into the fluorogenic dye within a 50 mL conical tube via a two-hole stopper. Regarding the two Pasteur pipettes inserted into the respective 50 mL conical tube, the fine tip of one of the pipettes was manually broken (or shortened) before being inserted into the two-hole stopper; the fine tip of this pipette did not touch the fluorogenic dye. Next, the end of the same Pasteur pipette, the end usually attached to a rubber bulb, was connected to a vacuum using rubber tubing. Regarding the second Pasteur pipette inserted into the two-hole stopper on the 50 mL conical tube containing the dye, its fine tip was also shortened (via manual breaking), but not as much as the previously mentioned pipette (“shorter” Pasteur pipette). Moreover, the “longer” Pasteur pipette had its fine tip immersed within the fluorogenic dye inside the conical tube. Subsequently, the “shorter” Pasteur pipette was connected to a Fume Hood vacuum, and the “longer” Pasteur pipette was connected to the Puff Generator machine; specifically, rubber tubing was used for connecting the pipettes to the vacuum and Puff Generator. To be more specific, the ends of each pipette (the ends of lime glass Pasteur pipettes which are usually connected to a rubber bulb) were connected to the rubber tubing. Furthermore, the entirety of the puffing protocol for each vape bar and control was conducted in a fume hood; additionally, surrounding lights were turned off to reduce exposure of the fluorogenic dye to light. Furthermore, each 50 mL conical tube containing 10 mL of fluorogenic dye was wrapped with aluminum foil to minimize the dye’s exposure to light. A red light was used to see whether the vape bar generated aerosols were indeed being bubbled through the fluorogenic dye; this is due to H_2_DCF-DA not absorbing red light.

In conjunction with the aforementioned puff generation set-up, once a vape bar was inserted into the Buxco Puff Generator, aerosol was generated and bubbled into the fluorogenic dye under a specific puff profile regiment. Under the particular puff regiment used in the study, a total of 20 puffs was generated through the Puff Generator apparatus; the puffing frequency was two puffs/min, and each puff had a volume of 55 mL and lasted 3.0 s. Different components making up the interior of the Puff Generator (the artificial lung, inhalation actuator, and exhalation actuator) worked together simultaneously to smoke the vape bar to the puff regiment inputted by the user. Further, the Puff Generator smoked each vape bar for ten minutes; the resulting aerosols then traveled from the tubing attached to the Puff Generator to the Pasteur pipette inserted into the 50 mL conical tube. Moreover, once ten minutes of a specific puff regiment had passed for one particular vape bar, the 50 mL conical tube containing the dye which had just been bubbled through with the aerosol of that specific vape bar was inverted several times and then put in ice. Additionally, tubing which connected the Puff Generator to the 10 mL fluorogenic dye within a respective 50 mL conical tube was rinsed with 70% Ethanol and then sterile ddH_2_O in between replicates for a bar of a specific flavor, vendor, and nicotine concentration and in between puffing regiments for different vape bars. After bubbling all vape bars in duplicates, each resulting fluorogenic dye sample was given 15 min to react within a 37 °C degree water bath (VWR 1228 Digital Water Bath); the resulting solution was then immediately analyzed via fluorescence spectroscopy.

### 2.3. Generation of Aerosols from Propylene Glycol: Vegetable Glycerin (PG:VG) Solutions, Negative Controls, and Positive Controls

The same puff generator device and puffing regiment used for bubbling the aerosols generated from the vape bars analyzed were used when bubbling solutions consisting of Propylene Glycol (PG) (Sigma-Aldrich, St. Louis, MO, USA) (Cat # P4347) and Vegetable Glycerin (VG) (Sigma-Aldrich, St. Louis, MO, USA) (Cat # G5516). In other words, a PG:VG control (humectant control) was used in conjunction with our vape bar analyses. To further explain, a PG:VG solution was prepared in a 15 mL conical tube; PG and VG were added together in a 1:1 ratio. Subsequently, the prepared PG:VG solution was vortexed for one minute, inverted several times, and then left on a laboratory shaker (Labnet, Edison, NJ, USA) (Mo: Gyrotwister GX-1000) at ten revolutions per minute (10 rpm) overnight before being used in an acellular ROS assay the following day. On the day of the acellular ROS analysis, 700μL of the PG:VG solution was pipetted into a new empty refillable JUUL pod with a 1.8 Ohm cotton wick atomizer (OVNStech, Shenzen, GD, China) (Mo: WO1 JUUL Pods). Subsequently, the PG:VG solution was allowed to sit in the pod for three to five minutes before being inserted into a rechargeable e-cigarette device (JUUL Labs Inc., Washington, DC, USA) (Mo: Rechargeable JUUL Device w/USB charger). Next, the JUUL device was inserted into the Puff Generator and was smoked under the same puff regiment as the disposable vape bars which were analyzed. Similar to the 1.8 Ohm coil PG:VG control described, the same process was used with a refillable cartridge using a 1.6 Ohm coil; in this case, Eleaf Elven pod cartridges (Eleaf Elven, Shenzen, GD, China) (Mo: Eleaf Elven Pod Cartridge) were used and inserted into a different rechargeable e-cigarette device (Eleaf Elven, Shenzen, GD, China) (Mo: Eleaf Elven Pod System).

For our negative control, air was bubbled through the fluorogenic dye; this was achieved by using the Puff Generator under the same puffing regiment as before but without inserting a disposable vape bar into the machine. For our positive control, cigarette smoke generated through burning conventional research cigarettes (Kentucky Tobacco Research & Development Center in the University of Kentucky, Lexington, KY, USA) (Mo: 3R4F) was bubbled through the fluorogenic dye. Also, the fluorogenic dye through which the 3R4F research cigarette smoke was bubbled through was diluted four-fold with freshly made dye. Each control (PG:VG heated with a 1.6 Ohm coil, PG:VG heated with a 1.8 Ohm coil, air, and the 3R4F cigarette) was run in duplicates. 

### 2.4. Fluorescence Spectroscopy and ROS Quantification

After bubbling aerosols from every vape bar during a specific day in which an acellular ROS assay was conducted, 100 μL of each prepared standard and each bubbled dye solution was added to 3.0 mL of fluorogenic dye.Further, 3.0 mL of dye was first added to a 16 × 100 mm Durex Borosilicate Glass culture tube (VWR) (Cat #: 47729-576), and then 100 μL of the bubbled dye solution and each standard was individually added to these culture tubes. Next, each culture tube was vortexed gently. Subsequently, each culture tube was placed within a 37 °C water bath for 15 min. Further, during the 15-min incubation period, surrounding lights were turned off, and only red lights were used. Afterward, standards were measured on a spectrofluorometer (Thermo Fisher Scientific, Waltham, MA, USA) (Mo. FM109535) in fluorescence intensity units (FIU); the same was carried out with the fluorogenic dye samples through which vape bar aerosols were bubbled; all of which was performed using the previously mentioned culture tubes. Additionally, readings displayed on the fluorometer (concentration in μM) were based on the generated hydrogen peroxide standard curve and measured as hydrogen peroxide, H_2_O_2_ equivalents.

### 2.5. Statistical Analysis

Statistical analyses of significance were calculated using one-way ANOVA as well as Tukey’s post-hoc test for multiple pair-wise comparisons by GraphPad Prism Software version 8.1.1. Samples were run in duplicates and experiments were repeated until consistent data were obtained. The results are shown as mean ± SEM with duplicates analyses. Data were considered to be statistically significant for *p* values < 0.05.

## 3. Results

### 3.1. Total ROS Concentration within Aerosols Generated from Vape Bars Vary by Flavor

**Figure 2:**
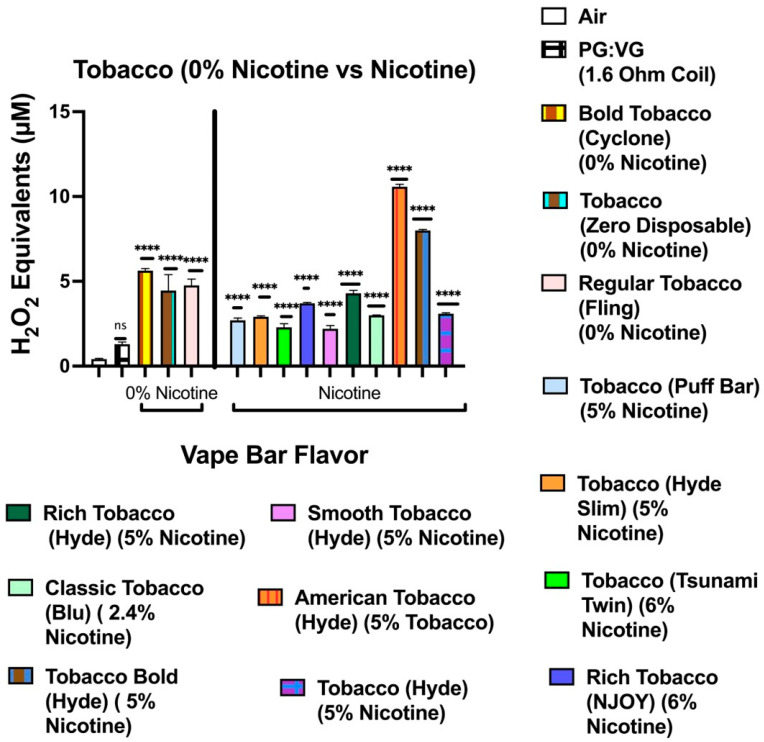
Generation of ROS by different tobacco-based flavors from various vendors. Acellular ROS was measured using a hydrogen peroxide standard within aerosols generated from various tobacco flavored disposable e-cigarette devices. Acellular ROS was also measured from the 1:1 ratio PG:VG control used. Each tobacco-based vape bar’s flavor, brand, and nicotine concentration are listed and color-coded. The resistance of the coil used to heat and aerosolize the PG:VG solution is also listed. All flavors and PG:VG controls listed on the graph above were compared to the control value of air. Data are represented as mean ± SEM, and significance was determined by one-way ANOVA. **** *p* < 0.0001 versus air controls. ns is abbreviated for “Non-Significant” versus air-controls (*p* > 0.05).

**Figure 3:**
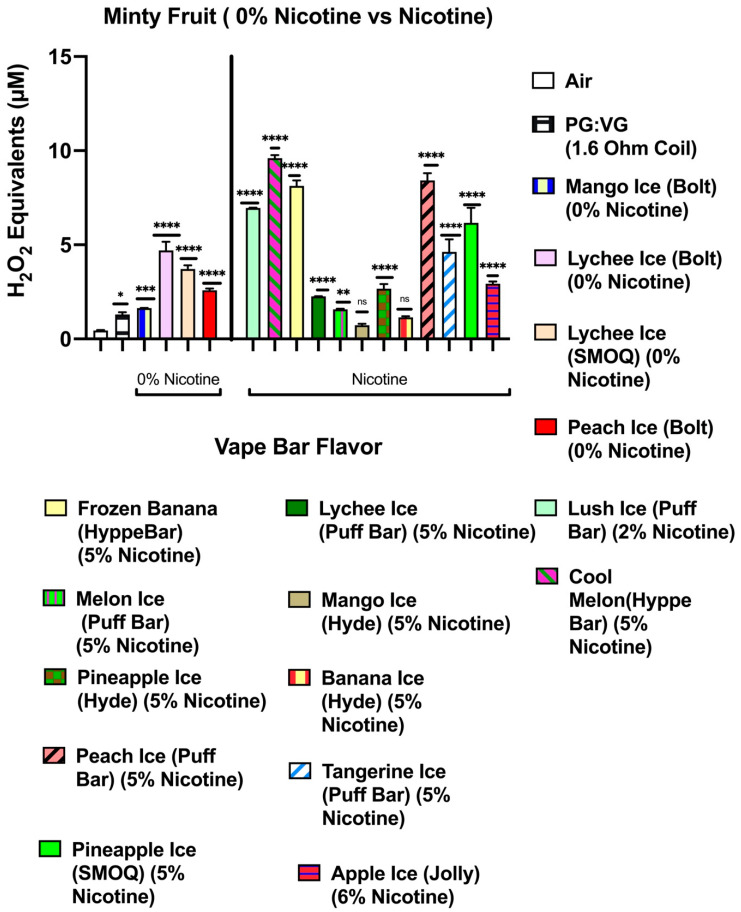
Generation of ROS by different minty fruit flavors from various vendors. Acellular ROS was measured from aerosols generated from various different minty fruit flavored disposable e-cigarette devices using a hydrogen peroxide standard. Acellular ROS was also measured from the 1:1 ratio PG:VG control used. Each minty fruit-based vape bar’s flavor, brand, and nicotine concentration are listed and color-coded. The resistance of the coil used to heat and aerosolize the PG:VG solution is also is also listed. All flavors and PG:VG controls listed on the graph above were compared to the control value of air. Data are represented as mean ± SEM, and significance was determined by one-way ANOVA. * *p* < 0.05, ** *p* < 0.01, *** *p* < 0.001, and **** *p* < 0.0001 versus air controls. ns is abbreviated for “Non-Significant” versus air controls (*p* > 0.05).

**Figure 4:**
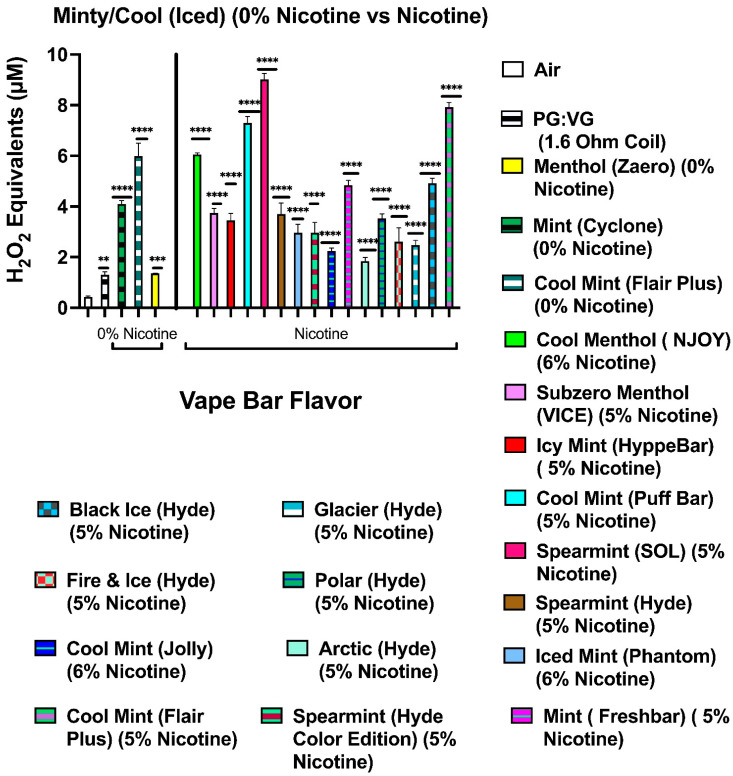
Generation of ROS by different Minty/Cool (Iced) flavors from various vendors. Acellular ROS was measured from aerosols generated from various different minty/cool (iced)-flavored disposable e-cigarette devices using a hydrogen peroxide standard. Acellular ROS was also measured from the 1:1 ratio PG:VG control used. Each Minty/Cool (Iced)-based vape bar’s flavor, brand, and nicotine concentration are listed and color-coded. The resistance of the coil used to heat and aerosolize the PG:VG solution is also listed. All flavors and PG:VG controls listed on the graph above were compared to the control value of air. Data are represented as mean ± SEM, and significance was determined by one-way ANOVA. Data are represented as mean ± SEM, and significance was determined by one-way ANOVA. ** *p* < 0.01, *** *p* < 0.001, and **** *p* < 0.0001 versus air controls. ns is abbreviated for “Non-Significant” versus air controls (*p* > 0.05).

**Figure 5:**
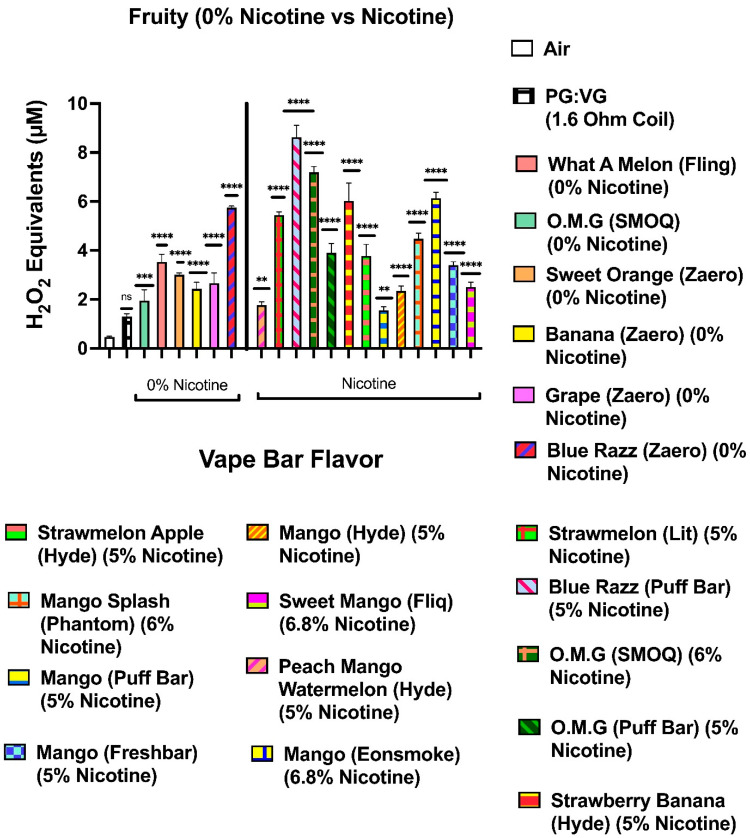
Generation of ROS by different fruity flavors from various vendors. Acellular ROS was measured from aerosols generated from various fruit-flavored disposable e-cigarette devices using a hydrogen peroxide standard. Acellular ROS was also measured from the 1:1 ratio PG:VG control used. Each fruity-based vape bar’s flavor, brand, and nicotine concentration are listed and color-coded. The resistance of the coil used to heat and aerosolize the PG:VG solution is also listed. All flavors and PG:VG controls listed on the graph above were compared to the control value of air. Data are represented as mean ± SEM, and significance was determined by one-way ANOVA.** *p* < 0.01 *** *p* < 0.001, and **** *p* < 0.0001 versus air controls. ns is abbreviated for “Non-Significant” versus air controls (*p* > 0.05).

**Figure 6:**
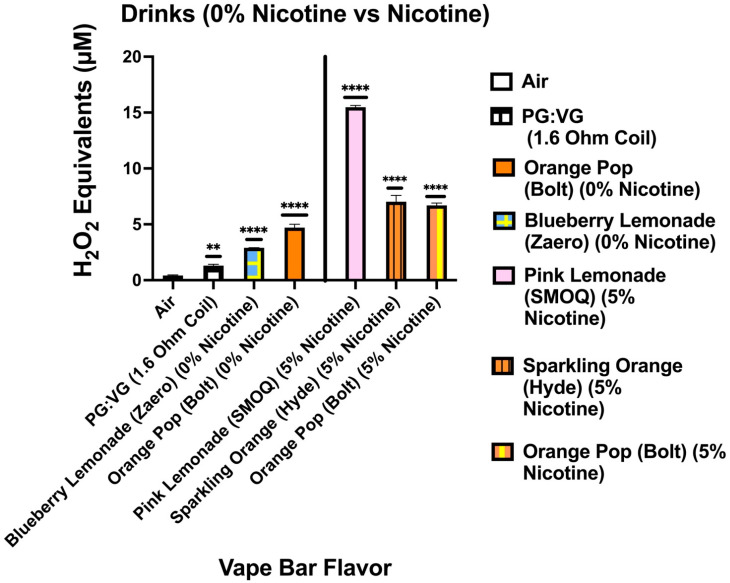
Generation of ROS by different drink flavors from various vendors. Acellular ROS was measured from aerosols generated from various drink flavored disposable e-cigarette devices using a hydrogen peroxide standard. Acellular ROS was also measured from the 1:1 ratio PG:VG control used. Each drink-based vape bar’s flavor, brand, and nicotine concentration are listed and color-coded. The resistance of the coil used to heat and aerosolize the PG:VG solution is also listed. All flavors and PG:VG controls listed on the graph above were compared to the control value of air. Data are represented as mean ± SEM, and significance was determined by one-way ANOVA. ** *p* < 0.01 and **** *p* < 0.0001 versus air controls. ns is abbreviated for “Non-Significant” versus air controls (*p* > 0.05).

**Figure 7:**
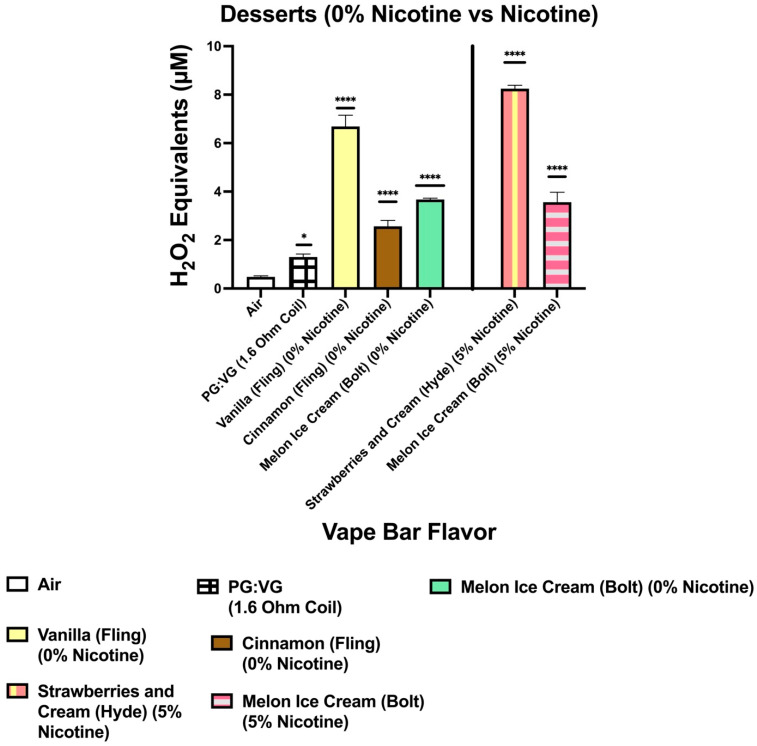
Generation of ROS by different dessert flavors from various vendors. Acellular ROS was measured from aerosols generated from various dessert flavored disposable e-cigarette devices using a hydrogen peroxide standard. Acellular ROS was also measured from the PG:VG control used. Acellular ROS was also measured from the 1:1 ratio PG:VG control which was used. Each dessert-based vape bar’s flavor, brand, and nicotine concentration are listed and color-coded. The resistance of the coil used to heat and aerosolize the PG:VG solution is also listed. All flavors and PG:VG controls listed on the graph above were compared to the control value of air. Data are represented as mean ± SEM, and significance was determined by one-way ANOVA. * *p* < 0.05 and **** *p* < 0.0001 versus air controls. ns is abbreviated for “Non-Significant” versus air controls (*p* > 0.05).

Our data show that aerosols generated from disposable flavored vape bars produced differential H_2_O_2_ equivalents. The aerosols generated from different flavored vape bars contained significantly different total ROS concentrations (μM H_2_O_2_) (Figure 2, Figure 3, Figure 4, Figure 5, Figure 6 and Figure 7). The disposable vape bars with the highest ROS content within each of the six previously mentioned flavor categories (Tobacco, Minty Fruit, Minty/Cool (Iced), Fruity, Drinks/Beverages, and Desserts) were Hyde American Tobacco (5% nicotine), Hyppe Bar: Cool Melon (5% nicotine), NJOY: Cool Menthol (6% nicotine), Puff Bar: Blue Razz (5% nicotine), SMOQ: Pink Lemonade (5% nicotine), and Strawberries and Cream (5% nicotine), respectively (Figure 2, Figure 3, Figure 4, Figure 5, Figure 6 and Figure 7). The aerosol produced by the 5% nicotine Hyde American Tobacco flavored bar contained 10.43–10.72 µM H_2_O_2_ (Figure 2), the aerosol produced by the 5% nicotine Hyppe Bar Cool Melon bar was 9.44–9.76 µM H_2_O_2_ (Figure 3), and the aerosol generated from the 5% Puff Bar Blue Razz contained a ROS content of 8.15–9.11 µM H_2_O_2_ (Figure 5). Moreover, the ROS content within the aerosols generated by SOL: Spearmint (5% nicotine), SMOQ: Pink Lemonade (5% nicotine), Strawberries and Cream (5% nicotine), was 8.78–9.25 µM H_2_O_2_, 15.32–15.63 µM H_2_O_2_, and 8.11–8.39 µM H_2_O_2_, respectively (Figure 4, Figure 6 and Figure 7, respectively). Among the fruity-flavored vape bars analyzed, ROS levels generated from the 0 and 5% nicotine-containing Blue Razz bars were the highest among every 0% nicotine-containing fruity-flavored bar (5.68–5.82 µM H_2_O_2_) and every nicotine-containing fruity-flavored bar (8.15–9.11 µM H_2_O_2_), respectively (Figure 5). Additionally, the highest ROS content among all vape bars analyzed in this experiment was found within the aerosol generated by the 5% nicotine-containing SMOQ: Pink Lemonade vape bar (15.32–15.63 µM H_2_O_2_) under the “Drinks/Beverages” flavor category (Figure 6).

Among the 0% nicotine vape bars analyzed, bars which generated aerosols containing the highest ROS content within the Tobacco, Minty Fruit, and Minty/Cool (Iced) flavor categories were Cyclone’s Bold Tobacco flavored-bar (0% nicotine), Bolt’s Lychee Ice flavored-bar (0% nicotine), and Flair Plus’s Cool Mint flavored-bar (0% nicotine), respectively (Figure 2, Figure 3 and Figure 4). Additionally, the 0% nicotine bars which generated aerosols containing the highest ROS content within the Fruity, Drink, and Dessert flavor categories were Zaero’s Blue Razz flavored bar (0% nicotine), Bolt’s Orange Pop flavored bar (0% nicotine), and Fling’s Vanilla flavored bar (0% nicotine) (Figure 5, Figure 6 and Figure 7).

### 3.2. Total ROS Concentration in Aerosols Generated by Identical Flavored Vape Bars Vary with Nicotine Concentration

**Figure 8:**
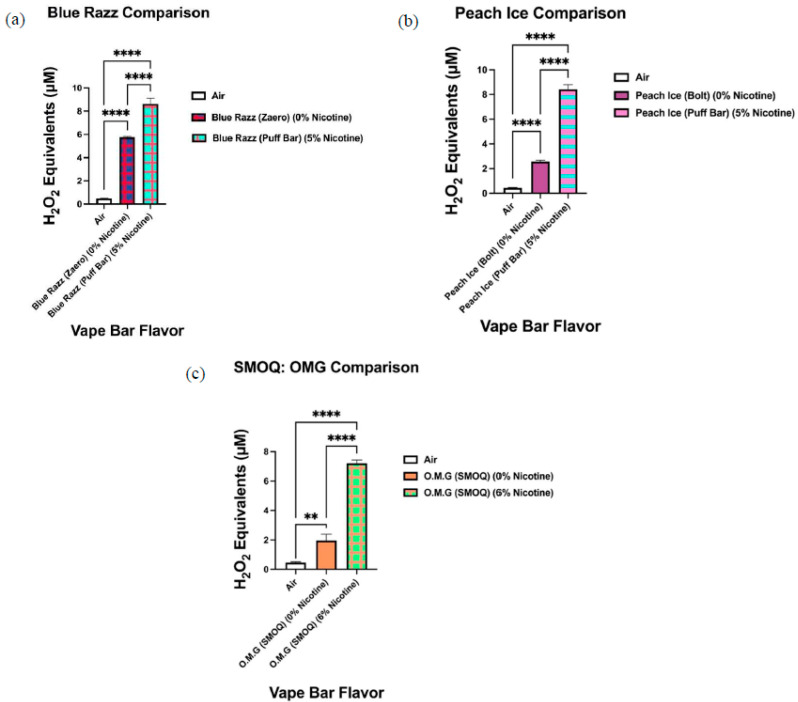
Direct relationship between ROS generation and nicotine concentration within aerosols generated from Blue Razz (**a**), Peach Ice (**b**), and O.M.G (**c**) flavored vape bars. Acellular ROS was measured within aerosols generated from disposable e-cigarettes of the same flavor but different nicotine concentrations using a hydrogen peroxide standard. The disposable vape bars that are shown above within each graph (**a**,**b**) are of the same specific flavor, but each bar was manufactured from a different vendor; these flavors are Blue Razz (**a**) and Peach Ice (**b**). The O.M.G flavored vape bars of differing nicotine concentrations which were analyzed were manufactured from the same vendor (SMOQ) (**c**). Names of each vape bar’s flavor, its brand, and its respective nicotine concentration are listed on the side of each respective graph. Pairwise comparisons consisted of those between ROS generated from vape bars and those of other vape bars as well as with the air control. Data are represented as mean ± SEM, and significance was determined using a one-way ANOVA. ** *p* < 0.01 and **** *p* < 0.0001 versus air controls and for specific pairwise comparisons.

**Figure 9:**
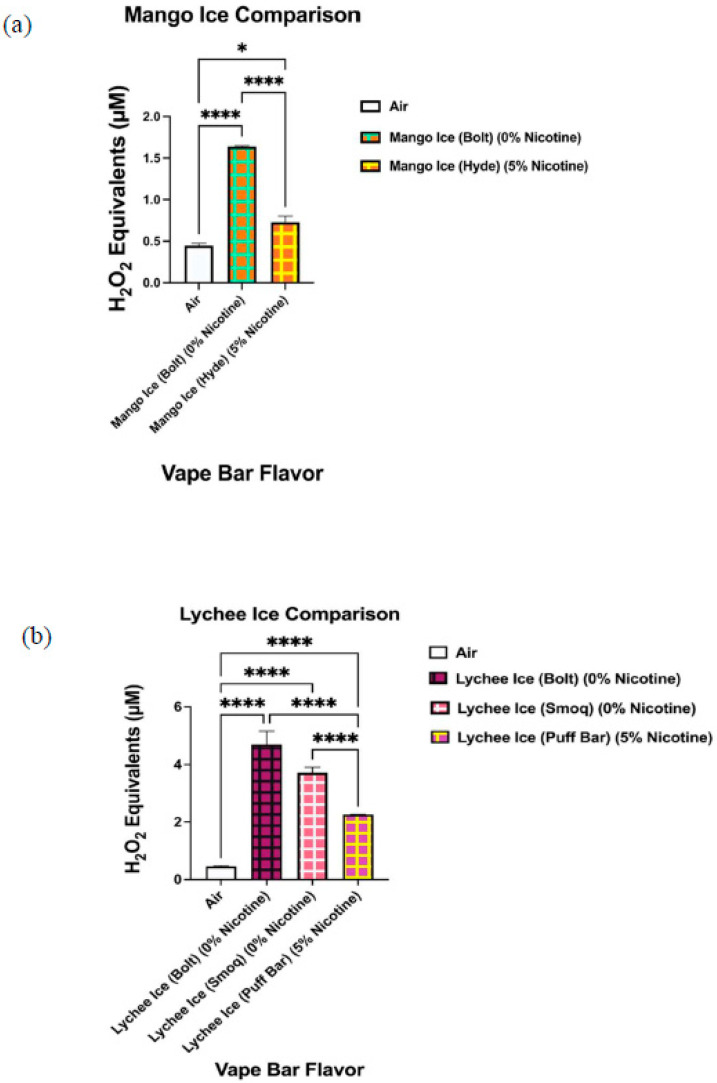
Inverse relationship between ROS generation and nicotine concentration in aerosols generated from Mango Ice (**a**) and Lychee Ice (**b**) flavored disposable e-cigarettes. Acellular ROS was measured using a hydrogen peroxide standard within aerosols generated from disposable e-cigarettes of the same flavor but different nicotine concentrations. Regarding disposable vape bars that were of the same specific flavor (Mango Ice (**a**) and Lychee Ice (**b**)), each vape bar was manufactured from a different vendor. The names of each vape bar’s flavor, its brand, and its respective nicotine concentration are listed on the side of each respective graph. Pairwise comparisons consisted of those between aerosols generated from vape bars and other vape bars as well as with air. All flavors were compared to the control value of air and to other flavors. Data are represented as mean ± SEM, and significance was determined by one-way ANOVA. * *p* < 0.05 and **** *p* < 0.0001 for specific pairwise comparisons.

**Figure 10:**
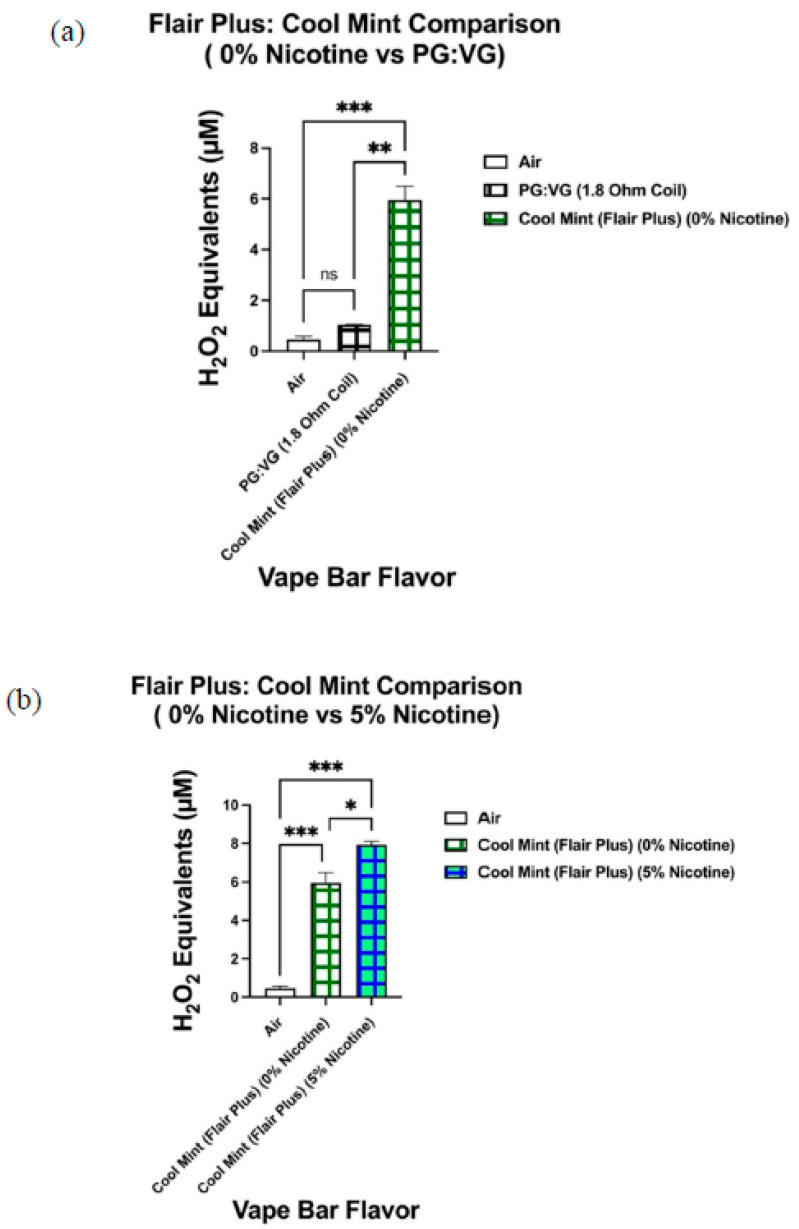
Differential ROS levels among aerosols produced from Cool Mint flavored vape bars of varying nicotine concentrations manufactured by Flair Plus. Acellular ROS was measured using a hydrogen peroxide standard within aerosols generated from vape bars of varying concentrations of nicotine (0 and 5%) of the same flavor and vendor (Flair Plus and Cool Mint), respectively. The corresponding 0% nicotine-containing bar was compared to a PG:VG control; this PG:VG control contained the same PG:VG ratio and was heated using a coil of the same resistance as the vape bar shown above (**a**). Another comparison in ROS concentration was made between the aerosols generated from the 0% nicotine containing Flair Plus: Cool Mint bar and the 5% nicotine-containing Flair Plus: Cool Mint bar (**b**). The name of each vape bar’s flavor, its brand, and its respective nicotine concentration are listed on the side of each respective graph; the same labeling method was used for the PG:VG control analyzed. Data are represented as mean ± SEM, and significance was determined using a one-way ANOVA. * *p* < 0.05, ** *p* < 0.01, and *** *p* < 0.001 for specific pairwise comparisons shown above. ns is abbreviated for “Non-Significant” versus air controls (*p* > 0.05).

**Figure 11:**
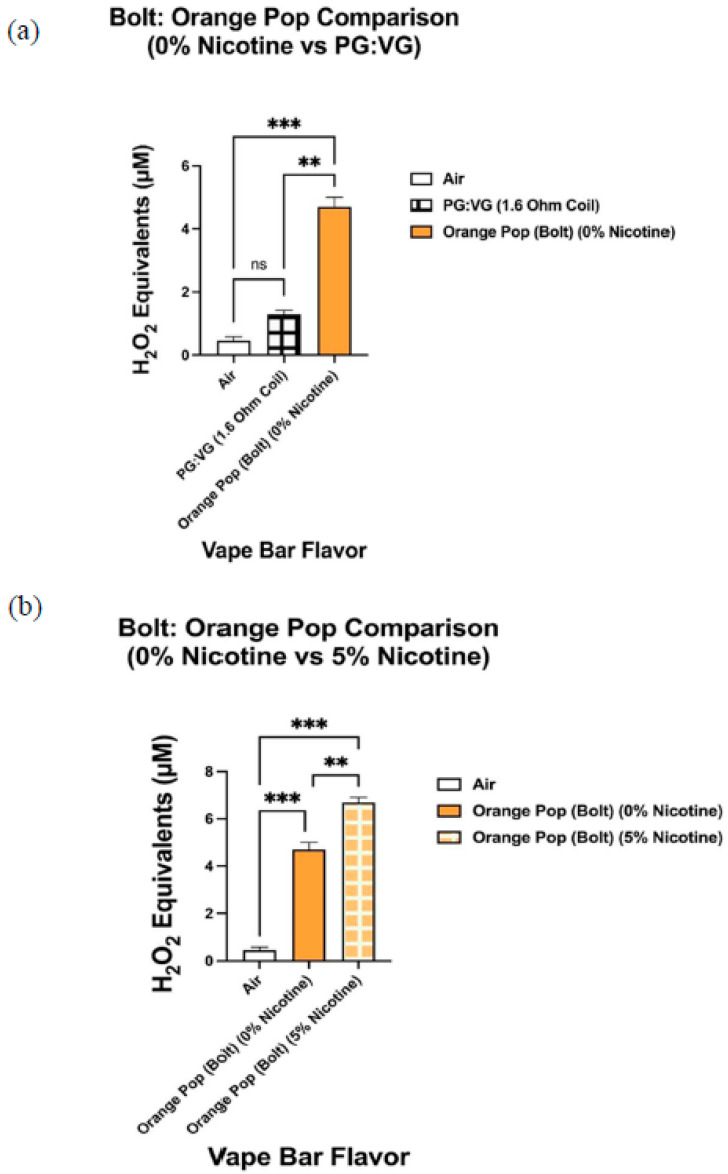
Differential ROS levels among aerosols produced from Orange Pop flavored vape bars of varying nicotine concentrations manufactured by Bolt. Acellular ROS was measured using a hydrogen peroxide standard within aerosols generated from vape bars of varying nicotine concentrations which were of the same flavor and vendor (Orange Pop and Bolt, respectively). The corresponding 0% nicotine-containing bar was compared to a PG:VG control; this PG:VG control contained the same PG:VG ratio and was heated using a coil of the same resistance as the vape bar shown above (**a**). Another comparison in ROS concentration was made between the aerosols generated from the 0% nicotine-containing Bolt: Orange Pop bar and the 5% nicotine-containing Bolt: Orange Pop (**b**). The name of each vape bar’s flavor, its brand, and its respective nicotine concentration are listed on the side of each respective graph; the same labeling method was used for the PG:VG control analyzed. Data are represented as mean ± SEM, and significance was determined using a one-way ANOVA. ** *p* < 0.01and *** *p* < 0.001 for specific pairwise comparisons shown above. ns is abbreviated for “Non-Significant” versus air controls (*p* > 0.05).

**Figure 12:**
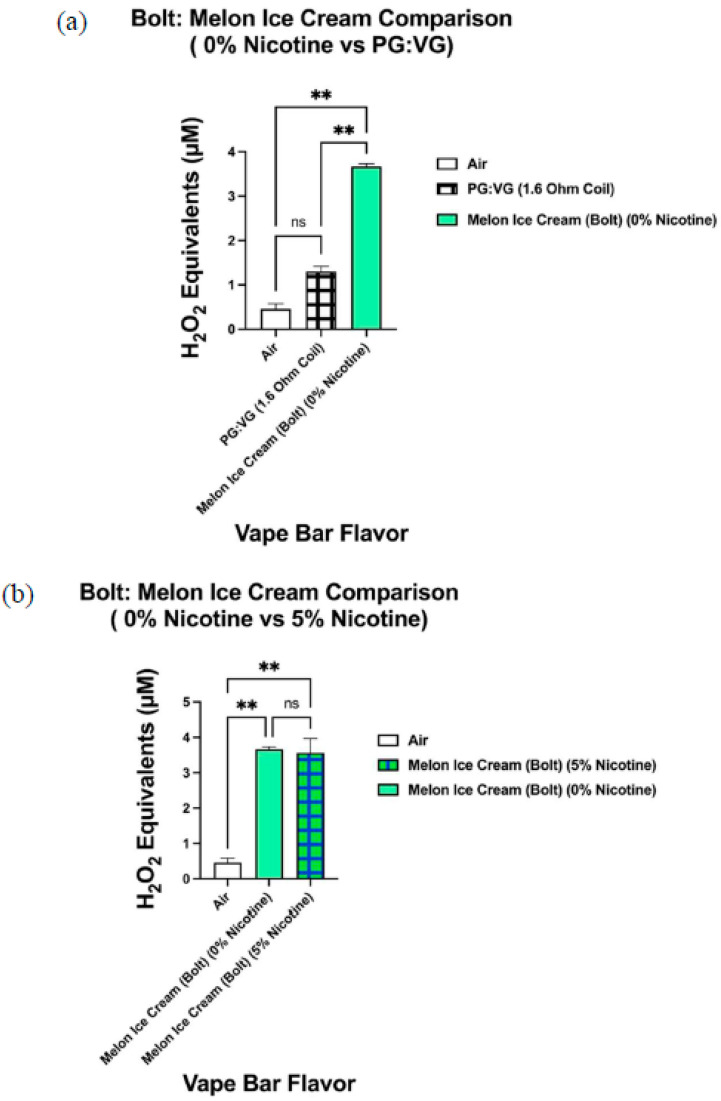
Differential ROS levels among aerosols generated from Melon Ice Cream flavored vape bars of varying nicotine concentrations manufactured by Bolt. Acellular ROS was measured using a hydrogen peroxide standard within aerosols generated from vape bars of various nicotine concentrations (0 and 5%) of the same flavor and vendor (Melon Ice Cream and Bolt, respectively). The corresponding 0% nicotine-containing bar was compared to a PG:VG control; this PG: VG control contained the same PG:VG ratio and was heated using a coil of the same resistance as the vape bar shown above (**a**). Another comparison in ROS concentration was made between the aerosols generated from the 0% nicotine-containing Bolt: Melon Ice Cream bar and the 5% nicotine-containing Bolt: Melon Ice Cream (**b**). The name of each vape bar’s flavor, its brand, and its respective nicotine concentration are listed on the side of each respective graph; the same labeling method was used for the PG:VG control analyzed. Data are represented as mean ± SEM, and significance was determined using a one-way ANOVA. ** *p* < 0.01 for specific pairwise comparisons shown above. ns is abbreviated for “Non-Significant” versus air controls (*p* > 0.05).

Comparatively, we observed significant variations in generated ROS levels among identically flavored disposable vape bars of varying nicotine concentrations. The variations in ROS levels among identically flavored vape bars with different nicotine concentrations were observed for eight specific flavors (Blue Razz, Mango Ice, Peach Ice, Lychee Ice, Cool Mint, Orange Pop, Melon Ice Cream, and O.M.G (Orange, Mango, and Guava)) (Figure 8, Figure 9, Figure 10, Figure 11 and Figure 12). When analyzing ROS content produced from aerosols generated by Blue Razz flavored vape bars, we found that the aerosol generated by the nicotine-containing bar (5% nicotine) had significantly higher ROS than the respective non-nicotine-containing bar (0% nicotine) (Figure 8a). Likewise, we found that the aerosol generated by the nicotine-containing (5% nicotine) Peach Ice bar contained a significantly higher ROS content than that produced from a non-nicotine-containing Peach Ice bar (0% nicotine) (Figure 8b). In contrast, for both the Mango Ice and Lychee Ice flavors, we found that the aerosol generated from the non-nicotine-containing bar generated a significantly higher level of ROS than its respective nicotine-containing counterpart (Figure 9).

When analyzing the ROS content within aerosols produced by vape bars of the same flavor and vendor (Bolt, Flair Plus, and SMOQ, etc.), we observed significant variations in ROS levels among bars of varying nicotine concentrations (Figure 10, Figure 11 and Figure 12). Further, the ROS concentration within aerosol generated from the 0% nicotine Cool Mint bar from Flair Plus, 5.40–6.50 µM H_2_O_2_, was significantly lower than that within the aerosol generated from its corresponding 5% nicotine bar, 7.73–8.11 µM H_2_O_2_ (Figure 10b). Regarding the two Orange Pop vape bars made by Bolt, which we analyzed, aerosol within the corresponding 0% nicotine bar contained a ROS concentration of 4.39–5.00 µM H_2_O_2_, which is significantly lower than the ROS concentration within the aerosol generated from its respective 5% nicotine bar, 6.48–6.90 µM H_2_O_2_ (Figure 11b). Similarly, like Orange Pop, Melon Ice Cream is another flavor manufactured by Bolt at both 0% nicotine and 5% nicotine. Regarding the semi-quantification of ROS within the aerosols produced by the Melon Ice Cream flavored bar from Bolt, the ROS concentration within Bolt’s 0% nicotine Melon Ice Cream bar is 3.61–3.73 µM H_2_O_2_ (Figure 12b). Likewise, 3.61–3.73 µM H_2_O_2_ is not significantly different from the ROS concentration within the aerosol generated from the corresponding 5% nicotine bar, 3.16–3.97 µM H_2_O_2_ (Figure 12b). Regarding other comparisons of semi-quantified ROS levels within aerosols generated from vape bars of varying nicotine concentrations of the same flavor and vendor, SMOQ’s O.M.G bars were also analyzed (Figure 8c). The ROS concentration within the aerosol generated from SMOQ’s 6% nicotine O.M.G bar (6.96–7.43 µM H_2_O_2_) was significantly higher than that within the aerosol generated from the corresponding 0% nicotine bar (1.5–2.4 µM H_2_O_2_) (Figure 8c).

Regarding the non-nicotine-containing Flair Plus: Cool Mint bar (0% nicotine), the non-nicotine-containing Bolt: Orange Pop bar (0% nicotine), and the non-nicotine-containing Bolt: Melon Ice Cream bar (0% nicotine), there were significant differences between ROS levels within each aerosol generated from each of the three bars and the ROS levels within their respective aerosolized PG:VG controls. To further explain, the ROS content within the aerosol generated from Flair Plus’s 0% nicotine Cool Mint bar (5.40–6.50 µM H_2_O_2_) was significantly higher than that within the aerosol generated from PG:VG solution aerosolized (1.03–1.06 µM H_2_O_2_). To clarify, this specific PG:VG control aerosolized contains the same PG:VG ratio (1:1) and was heated using a coil of the same resistance as that used in the Flair Plus: Cool Mint bar (0% nicotine), 1.8 Ohms (Figure 10a).

Likewise, the ROS concentration within aerosol generated from the 0% nicotine Bolt: Orange Pop bar (4.39–5.00 µM H_2_O_2_) was significantly higher than that within the aerosol generated from the PG:VG solution vaporized using a 1.6 Ohm coil (1.18–1.42 µM H_2_O_2_) (Figure 11a). The resistance of the coil used in all Bolt disposable bars is 1.6 Ohms. Similarly, the respective PG:VG control used in the subsequent pairwise comparison with the 0% nicotine Bolt: Orange Pop bar (Figure 11a) had the same ratio of PG and VG as the respective bar (1:1). Regarding the non-nicotine-containing Bolt: Melon Ice Cream bar, the ROS concentration within the aerosolized 0% nicotine bar (3.61–3.73 µM H_2_O_2_) was significantly higher than that generated from the PG:VG control (1.18–1.42 µM H_2_O_2_) (Figure 12a). Again, to clarify, this specific PG:VG control, used in the aforementioned pairwise comparison, contains the same PG:VG ratio (1:1) and was heated using a coil of the same resistance as that used in the Bolt: Melon Ice Cream bar (0% nicotine), 1.6 Ohms.

## 4. Discussion

When analyzing the ROS content emitted by vape bars within each flavor category (Tobacco, Fruity, Minty Fruit, Minty/Cool (Iced), Drinks/Beverages, and Desserts), we observed differential ROS production among the different flavored bars. In addition, within each of the six flavor categories analyzed, different flavored disposable vape bars with the same nicotine content produced variable levels of ROS relative to the respective air control. The Tobacco, Fruity, Minty Fruit, Minty/Cool (Iced), Drinks/Beverages, and Dessert flavor categories with and without nicotine were selected for our analyses due to the popularity of these flavor categories among e-cigarette users, especially among e-cigarette users in middle and high school, after the FDA’s 2020 e-cigarette flavoring enforcement policy [1]. Furthermore, the FDA’s 2020 flavoring enforcement policy prohibits companies from selling cartridge-based e-cigarettes with dessert, candy, fruit, mint flavors with nicotine, as well as any flavor excluding tobacco or menthol [1]. Any flavors without nicotine (zero nicotine) are still sold in the United States without any regulations, which are used in the present study.

Vape bars under the Minty Fruit flavor category were analyzed due to their recent rise in popularity among youth e-cigarette users. Further, around the same time that disposable e-cigarette sales surged following the FDA’s flavored e-cigarette enforcement policy on nicotine in 2020, a significantly high number of minty fruit e-cigarette flavors had entered marketplaces [15]. Further, the increased usage of minty (cool/iced) fruit flavors among e-cigarette users in the country necessitated us to analyze these flavors because of their potential to make further regulatory action more complicated due to Iced Fruit flavors not fitting into existing flavor categorizations [15]. Research investigating how flavoring chemicals affect ROS generation in e-cigarette generated aerosols has been explored minimally; however, a few recent studies have delved into the dependence that ROS generation from e-cigarettes may have on flavoring chemicals. Our study found that ROS levels generated from cigar/cigarillo smoke varied among different flavors [16]. Regarding studies conducted with e-cigarettes, another study found that ROS generation within the aerosols generated from cartridge-based e-cigarette devices was highly dependent on the vendor, puffing pattern, voltage, and the flavor of the cartridge-based e-cigarette device used [3]. Moreover, our lab’s previous study found that the flavorings used in e-liquids can induce an inflammatory response in monocytes; the study further found that this response is mediated through ROS production [17].

Additionally, we show that ROS content in aerosols generated in vape bars of identical flavors (Blue Razz, Peach Ice, Lychee Ice, Mango Ice, Orange Pop, Melon Ice Cream, Cool Mint, and O.M.G) varied among identically flavored bars of different nicotine content. However, only five out of eight flavors mentioned showed the corresponding nicotine-containing bar generating significantly higher ROS levels than its respective 0% nicotine-containing bar. Here, our data showed that the nicotine-containing Mango Ice and Lychee Ice bars contained significantly lower ROS levels than their corresponding 0% nicotine-containing bar. These results observed among the Mango Ice and Lychee Ice bars of differing nicotine content may have occurred because the pairwise comparisons between these identically flavored bars did not control for the vendor. In the pairwise comparisons between the 0% nicotine-containing Mango Ice bar and the 5% nicotine Mango Ice bar, each bar was made by a different manufacturer. Additionally, because each Mango Ice bar was manufactured by a different vendor, the PG:VG content within each bar may have also been different between the identically flavored bars. Previous studies have shown that the ratio of PG and VG used within an e-liquid led to significant alterations in ROS levels within generated aerosols. Similarly, when analyzing and comparing ROS content among the aerosols generated from the different Lychee Ice bars (0% nicotine and 5% nicotine-containing bars), each bar was made by a different vendor. Further, this means that among the Lychee Ice, Peach Ice, Mango Ice, and Blue Razz bars analyzed, pairwise comparisons between the 0% nicotine-containing bar and the 5% nicotine-containing bar did not control for the vendor. Similarly, this may explain the reason for a consistent relationship between increasing nicotine content and ROS generation is not seen among the Lychee Ice, Mango Ice, Peach Ice, and Blue Razz flavored bars. The ROS generated from the nicotine-containing Mango Ice and Lychee Ice bars was significantly lower than that within the aerosols generated from the corresponding 0% nicotine bar. In contrast, our data analyzing the Blue Razz and Peach Ice bars showed a direct relationship between increasing nicotine content and ROS production.

Consequently, to control for the vendor in determining how nicotine affects ROS generation, one could analyze the ROS content within aerosols generated by vape bars of the same flavor and vendor but of differing nicotine content. Correspondingly, we did this by determining the ROS concentration within aerosols generated from the Flair Plus: Cool Mint, Bolt: Orange Pop, Bolt: Melon Ice Cream, and SMOQ: O.M.G bars. Further, among three of the four vendor-specific flavors, we found that the aerosol from the respective nicotine-containing bar contained a significantly higher level of ROS than the corresponding 0% nicotine-containing bar. For instance, the 5% nicotine Flair Plus: Cool Mint bar generated an aerosol that contained a significantly higher level of ROS than that within its respective 0% nicotine bar; this suggests that nicotine contributes to this significant difference in ROS levels. Similar results were also observed when comparing the 0 and 5% nicotine-containing Orange Pop bars manufactured by Bolt and the O.M.G flavored bars (0 and 6% nicotine) manufactured by SMOQ. Further, we realized that to better elucidate the role nicotine in affecting ROS generated from flavored vape bars, more comparative acellular ROS analyses are needed between bars of the same specific flavor and vendor, but different nicotine concentrations. Further work is required to analyze more vape bars with differing nicotine concentrations made from the same vendor and of the same specific flavor.

Previous studies have shown that nicotine and the other constituents of e-liquids (flavoring agents, propylene glycol (PG), and vegetable glycerin (VG)) contribute to ROS production [5,18]. Similarly, Haddad et al. have shown that the ROS emission from aerosolized e-liquids was significantly affected by the PG:VG ratio of the e-liquid [19]. Propylene Glycol (PG) and Vegetable Glycerol (VG) are humectants, substances used to maintain moisture. Furthermore, PG and VG’s ability to attract and retain moisture allows e-cigarette users to feel what is known as a “throat hit”. A “Throat hit” refer to the sensation one who uses ENDS products feels in their throat caused by nicotine inhalation. Regarding one of the specific findings from Haddad Et al., the study found that increasing the percentage of VG within the base PG:VG liquid component of an e-liquid used within a rechargeable e-cigarette significantly increased ROS flux [19]. Similarly, another study by Bitzer et al. found increases in the PG content of an e-liquid used in rechargeable e-cigarettes led to heavy increases in free-radical production within the resulting aerosolized e-liquid [18].

These previous studies compelled us to determine the ROS concentration with the PG:VG base solution used within the vape bars analyzed in our study. We reasoned that by semi-quantifying the ROS content within the aerosols produced from the PG:VG component of vape bars we analyzed, the role flavoring chemicals and nicotine have in contributing to ROS production during e-liquid heating and aerosolization can be further elucidated. However, out of every vape bar analyzed in our study, the only two companies which provided the PG:VG content online were Bolt and Flair Plus; for both companies, the e-liquid component of the vape-bar contained a 1:1 ratio PG:VG solution. Accordingly, we prepared a 1:1 ratio PG:VG solution to be smoked and aerosolized using the Puff Generator in tandem with the other analyzed vape bars. Additionally, when looking into other specifications of the Flair Plus and Bolt bars analyzed, we saw that each vendor used a coil of a different resistance: 1.6 and 1.8 Ohms, respectively. Accordingly, we analyzed the ROS content within a 1:1 ratio PG:VG solution aerosolized using a 1.6 Ohm coil (via Eleaf Elven cartridges) and a 1.8 Ohm coil (via OVN: W01 JUUL cartridges).

Regarding how flavoring chemicals used in vape bars contribute to ROS emissions from vape bar-generated aerosols, research delving into how the interactions between different components of e-liquids contribute to ROS generation is lacking. However, a study by Son, Yeongkwon et al. found that the flavoring chemicals within flavoring agents (those including maltol, benzyl acetate, and anethole, etc.) may undergo redox cycling with transition metal ions found with e-liquids and produce ^•^OH [5]. Additionally, a previous study from our lab (Lerner et. al.) found that the oxidative nature of non-vaporized e-liquids is dependent on the flavoring additives used in an e-liquid [10]. For example, e-liquids containing fruity or sweet flavors were stronger oxidizing agents than corresponding tobacco flavored e-liquids [10]. Together, Lerner et al. findings and the present study suggest that flavoring chemicals themselves influence ROS production during e-liquid aerosolization. Our results comparing the ROS content within aerosols generated from different bars within each of the six major flavor categories (Tobacco, Minty Fruit, Minty/Cool (Iced), Fruity, Drinks/Beverages, and Desserts) suggest that ROS generation varies among different flavored bars. However, comparative acellular ROS analyses between a 0% nicotine-containing flavored vape bar and the PG:VG solution making up that same vape bar are needed to further investigate the role of flavoring agents in ROS production within vape bars. To further explain, a PG:VG solution heated and aerosolized using a coil of the same resistance as the flavored vape bars of interest is needed. By comparing the ROS generated between a 0% nicotine-containing flavored vape bar and an accurate PG:VG control, one can see whether the flavoring agents themselves play a role in changing the ROS levels generated upon a vape bar aerosolization.

Consequently, we conducted pairwise comparisons between ROS levels produced from three 0% nicotine-containing bars and their respective PG:VG controls. These three aforementioned 0% nicotine bars were manufactured by Flair Plus and Bolt; two of which were manufactured by Bolt and one of which was manufactured by Flair Plus. Next, when comparing the ROS content within the aerosol generated from the 0% nicotine Flair Plus: Cool Mint bar with that within the aerosol produced from its respective PG:VG control, the ROS content generated from the 0% nicotine bar was significantly higher than that within aerosolized PG:VG control. Moreover, the PG:VG ratio and the coil’s resistance used in the PG:VG control were the same as that used in the 0% nicotine-containing Flair Plus: Cool Mint bar; this specific pairwise comparison minimized PG:VG content and coil resistance as potential confounding influences. Accordingly, the previously mentioned results suggest that flavoring chemicals themselves (in particular the ones used to make the Cool Mint flavor) significantly contribute to ROS generation upon e-liquid heating and subsequent aerosolization.

Similarly, pairwise comparisons between the 0% nicotine Bolt: Orange Pop bar and its respective PG:VG control and between the 0% nicotine Bolt: Melon Ice Cream bar and its PG:VG control also suggest the same conclusion we reached upon our analysis of the 0% nicotine-containing Cool Mint bar from Flair Plus and its PG:VG control. To clarify Bolt disposable bars have a PG:VG ratio of 1:1 and use a 1.6 Ohm coil to heat their e-liquid component. Consequently, the PG:VG control used in the pairwise comparisons with the aforementioned Bolt 0% nicotine bars contained a PG:VG ratio of 1:1 and was aerosolized using a 1.6 Ohm coil. Subsequently, our data showed that both 0% Bolt bars (Orange Pop and Melon Ice Cream) contain a significantly higher ROS content than their corresponding PG:VG controls. These results further suggest that flavoring agents (in this case, the ones used to make Orange Pop and Melon Ice Cream flavors) significantly contribute to ROS generation by flavored vape bars.

Regarding a limitation of our study, the only PG:VG controls we used were those with a 1:1 ratio composition of both PG and VG. These were heated using 1.6- and 1.8-Ohm coils. PG:VG controls utilizing this specific ratio of PG and VG (1:1) and which were heated using 1.6- and 1.8-Ohm coils. This was used because the PG:VG ratio and the resistance of the coils used in the Flair Plus and Bolt bars we had analyzed. Flair Plus and Bolt were the only two commercial manufacturers of the disposable vape bars used in this study that provided information on their PG: VG content, coil resistance, and that manufactured both non-nicotine-containing and nicotine-containing bars. Further, we could not find the resistance of the coils used in many of the other vape bars we analyzed, nor could we find the PG:VG ratio used within those bars. Consequently, our PG:VG controls were modeled after the specifications of the Flair Plus and Bolt bars analyzed. Additionally, in our data comparing the ROS generated from every single vape bar within each of the six major flavor categories analyzed, we only included the PG:VG control heated using a 1.6 Ohm coil. We did this because information on the resistance of the coils used in many of the other vape bars included in this study was not provided by the respective vendors of those bars. Secondly, we realized that out of all the vape bars we analyzed whose vendors provided information on their coil resistance, the highest number of bars used a 1.6 Ohm coil. Consequently, to maintain consistency among the first six graphs provided in the paper, we only included the 1.6 Ohm PG:VG control within each of those six graphs. However, acellular ROS assays and comparative analyses between different flavored vape bars in future studies should only be conducted once the resistance of coils used in the vape bars one plans to analyze is known. This is because coil resistance is a key part of the heating and aerosolization process within ENDS [20], and possibly in vape bars. Accordingly, future related studies must include PG:VG controls that are aerosolized using coils of the same resistance of each vape bars analyzed in the respective study.

Similarly, regarding another limitation of this study, the only two commercial manufacturers of the vape bars we analyzed in our study that provided information on PG:VG ratios used in component e-liquids were Flair Plus and Bolt. Consequently, the PG:VG controls we used consisted only of a 1:1 ratio of PG:VG as these were the PG and VG ratios used in bars from Bolt and Flair Plus. The other commercial manufacturers of the vape bars we analyzed in our study did not provide information on the ratio of PG and VG contained in their vape bars. Consequently, we could not semi-quantify the ROS within aerosols produced from solutions of the same PG:VG ratio as those used in many of the vape bars we analyzed in this study. For these reasons, when producing graphs and including pairwise comparisons between the Blue Razz, Peach Ice, Mango Ice, and Lychee Ice bars of differing nicotine content, we did not include the ROS generated from our PG:VG controls. Our reasoning for this was because we did not know the ratio of the PG:VG used within the Blue Razz, Peach Ice, Mango Ice, and Lychee Ice bars; consequently, conducting pairwise comparisons between the PG:VG controls we used and each of the Blue Razz, Peach Ice, Mango Ice, and Lychee Ice bars of varying nicotine content would not have been scientifically sound. Furthermore, NMR spectroscopy using the e-liquids isolated from all the vape-bar we analyzed will determine each bar’s specific PG:VG ratio. In the future, when conducting acellular ROS analyses of flavored vape bars, we will use NMR spectroscopy to determine each bar’s PG:VG ratio to make an accurate PG:VG control for subsequent acellular ROS assays (both for bars whose manufactures provide information of PG:VG ratios and those which do not).

Moreover, assessing the ROS generation due to ‘cooling agents’ in ENDS is vital in determining the toxicity of vape bars with dual and multi flavors. Studies have found variations in the levels of synthetic cooling agents, such as WS-3 and WS-23, in cool (iced) flavors among e-cigarettes manufactured by various companies [21,22]. These cooling agents induce cytotoxicity in BEAS-2B lung epithelial cells, suggesting their adverse toxic effects upon inhalation [21]. Furthermore, future studies assessing the acellular ROS generation by cooling agents should consider the confounding factors, such as flavor category and nicotine concentration, as these constituents form secondary reactive species upon heating. Further, these future acellular ROS analyses must include a fruity-flavored vape bar (e.g., apple), its respective cool (iced) flavor (e.g., apple ice), an appropriate PG:VG control, and an appropriate salt nicotine control (using a PG:VG solvent) [23]. Additionally, acellular ROS assays conducted to investigate the effects cooling agents have in ROS generation from vape bars must include fruity flavored and respective cool (iced) flavored vape bars manufactured by various vendors. This may include flavored bars with or without nicotine (tobacco and mint/menthol flavors) [24].

Overall, our results suggest that different flavoring chemicals used in vape bars contribute to variations in the breakdown of the chemical bonds holding together the components of the e-liquid within a vape bar during thermal degradation, leading to differential ROS levels in generated aerosols. Additionally, our pairwise comparisons made between vape bars with different nicotine concentrations but the same specific flavor and vendor suggest nicotine itself has a role in influencing ROS generation within aerosolizing vape bars. In general, cool (iced) flavors generated differential ROS than their counterpart non-cool (iced) flavors. However, further assays are needed to elucidate how both the flavor of a vape bar and its corresponding nicotine concentration affect ROS generation within vape bars, and immune-inflammatory responses in mouse model as seen previously [25]. Future studies can use Gas Chromatography–Mass Spectrometry (GC–MS) to analyze the compounds within flavoring agents within flavored vape bars. For example, using GC-MS to analyze the e-liquids extracted from minty and cool (iced) vape bars can provide more insight on the cooling agents used within these specific flavored vape bars. In addition, Electron Paramagnetic Resonance (EPR) Spectroscopy can analyze the relative proportions of specific ROS (H_2_O_2_, O_2_^•^^−^, and ^•^OH) and free radicals within the aerosol generated from vape bars.

Future studies involving acellular ROS analyses using different flavored vape bars should also include a PG:VG control which includes nicotine (either free-base or nicotine benzoate). Further, when analyzing the ROS generated from vape bars of the same flavor and vendor but different nicotine concentrations, in addition to making a PG:VG control made up of the same ratio of PG and VG and heated using a coil of the same resistance as that used in the vape bars of interest, one can also make another control consisting of PG:VG and nicotine. Further, one can make a PG:VG control which includes the same percentage of nicotine salt used in the e-liquid component of their bars of interest. Subsequently, acellular ROS analyses among bars of the same flavor and vendor but different nicotine concentrations, a PG:VG control and PG:VG control with nicotine may show whether or not ROS generated from vape bars varies as a function of nicotine content. However, due limitations in our inventory, we could not produce a PG:VG w/nicotine control and aerosolize it to semi-quantify its ROS content.

## 5. Conclusions

Overall, our results concur with our initial hypothesis that ROS generated from disposable e-cigarette bars varies among different flavors and flavors of different nicotine content. The breakdown of chemical bonds holding together an e-liquid via thermal degradation leads to ROS production in generated aerosols. Further, any alterations in ROS production from e-liquids must arise due to changes in the breakdown of these chemical bonds during thermal degradation (in frequency, timing, etc.). Accordingly, our results seem to suggest that both flavoring agents and nicotine in some way alter the breakdown of chemical bonds holding together a vape bar’s component e-liquid.

Future studies are required to analyze a much higher number of flavored vape bars to better understand the relationship between nicotine and ROS generation and between flavoring chemicals and ROS generation within disposable e-cigarettes. Furthermore, in addition to analyzing a greater number of vape bars, more acellular ROS comparisons should be performed between vape bars that control for the vendor in multiple emerging flavors/vendors which are present in the market, thereby reducing the confounding influence a specific vendor may have on ROS generation. Additionally, for future studies analyzing the ROS generated by bars of the same specific flavor and vendor, corresponding PG:VG and PG:VG w/nicotine controls should be used for every vape bar analyzed.

Furthermore, the chemical constituents of a vape bar’s flavoring agents with differential cool (iced) flavors, and the quantities of specific free radicals within its generated aerosols can be determined through GC–MS and EPR Spectroscopy, respectively. These assays can be used to understand how the physicochemical interactions inside an e-liquid undergoing thermal degradation contribute to differential ROS generation among different flavors. Further, in conjunction with the recommended future studies, the results of our preliminary study can generate evidence used in favor of public health and regulatory policies that lead to the regulation of products, such as vape bars and other flavored/non-flavored ENDS.

## Data Availability

We declare that we have provided all the data in figures.
